# Editorial: Prediabetes and endocrine function

**DOI:** 10.3389/fendo.2023.1268552

**Published:** 2023-08-17

**Authors:** Andile Khathi, Sam Dagogo-Jack

**Affiliations:** ^1^ School of Laboratory Medicine and Medical Sciences, Discipline of Human Physiology, University of KwaZulu-Natal, Durban, South Africa; ^2^ Division of Endocrinology, Diabetes & Metabolism, The University of Tennessee Health Science Center, Memphis, TN, United States

**Keywords:** prediabetes, endocrine function, frequency and distribution of studies, biomarkers, surrogate markers

## Introduction

Type 2 diabetes mellitus (T2DM) is a condition characterized by chronic hyperglycaemia caused by insulin resistance or insulin insufficiency (1). T2DM accounts for approximately 90% of the cases worldwide and leads to a variety of microvascular and macrovascular complications (2). By 2040, it was predicted by the International Diabetes Federation (IDF) that around 642 million people worldwide are expected to be diagnosed with T2DM (2). The onset of T2DM is often preceded by an asymptomatic condition known as prediabetes where the blood glucose levels are above the normal range but below the threshold for the diagnosis of T2DM (3). Pre-diabetes is asymptomatic and has been shown to increasing in prevalence (3). The main risk factors that have been identified for this condition include chronic consumption of high calorie diets, obesity and sedentary lifestyles (4). Studies have shown that the intermediate hyperglycemia associated with prediabetes may result in chronic sub-clinical inflammation and increased generation of reactive oxygen species (ROS) which may in turn have consequences on metabolic function. Indeed, studies have shown that the complications that occur in T2DM begin in pre-diabetes (5–7). In this Research Topic, we compiled papers looking at the effects of prediabetes on endocrine function as well as looked at possible new biomarkers and surrogate markers for prediabetes.

## Frequency and distribution of studies on prediabetes

Prediabetes is defined as a condition of intermediate hyperglycaemia characterized by the impaired fasting glucose, impaired glucose tolerance and elevated levels of glycated haemoglobin (3). Prediabetes is a growing public health concern worldwide and this has prompted studies globally. This is evidenced by the bibliometric analysis done by Zhao and Li, they looked at worldwide trends of prediabetes from 1985 to 2022. The study highlighted research hotspots as well as development patterns in the field of prediabetes with a strong focus on global research outcomes. The study highlighted the increasing frequency of publications in this field over the last few decades. More significantly, the study showed the wide distribution in the journals where the work was published as well as the countries and universities where the work was being done.

## Effects on endocrine function

Several studies have previously shown that the complications often associated with type 2 diabetes actually begin prediabetes (8, 9). In this Research Topic, Naidoo et al., showed that there are derangements in calcium homeostasis due to compromised renal function during the prediabetic state. The study further showed that calcium-regulating organs compensate for renal calcium wastage and are aimed at maintaining normocalcaemia. The effects associated with prediabetes on calcium-regulating organs are directed towards promoting increased renal calcium reabsorption, increased renal vitamin D activation, increased intestinal calcium absorption and decreased bone resorption followed by increased bone formation. This was evidenced by increased expression of renal calcium transport markers and intestinal calcium transport markers in addition to increased osteocalcin and decreased deoxypyridinoline levels. This was supported by another research article in this topic by Liu et al.. This study analysed bone mineral density in initially normoglycaemic participants in the Pathobiology of Prediabetes in a Biracial Cohort (POP-ABC) study. This was in relation to the incidence of prediabetes during 5 years of follow-up. The main finding was that study participants who developed incident prediabetes during 5 years of follow-up tended to have higher baseline bone mineral density suggesting compensatory mechanisms in calcium homeostasis during prediabetes. Another study conducted by Krisnamurti et al. in a prediabetic rat model further provided evidence of the involvement of vitamin D in the development of prediabetes. Vitamin D deficiency has been frequently linked to insulin resistance and diabetes. In this study, the authors show that vitamin D supplementation reduces insulin resistance in prediabetic rats and that the reduction might be due to the effects of vitamin D on IRS, PPARg, and NF-kB expression. These studies collectively show both the effects of prediabetes on calcium homeostasis as well as how vitamin D supplementation could be used to reduce insulin resistance in the prediabetic state.

## Identification of biomarkers and surrogate markers

Various studies have embarked on identifying novel biomarkers and surrogate markers to assist with early identification and risk markers of prediabetes. Jiang et al. showed that higher visceral adiposity index values are positively associated with insulin resistance while Wang et al. showed that monitoring AST/ALT ratio could be beneficial as a predictor of insulin resistance in both males and females. Han et al. on the other hand demonstrated a positive non-linear relationship between the triglyceride glucose-BMI value and the risk of developing T2DM in patients that already have prediabetes. Yang et al. showed the use of a metabolite-based biomarker for early patient diagnosis and treatment of prediabetes while Al Akl et al. showed that the triglyceride-waist-height ratio may be a good marker in detecting prediabetes.

## Conclusion

Recent literature shows an upsurge in the study of prediabetes and the effects that it has on endocrine function. While there is still a lot more work to be done in this field, the latest studies have suggested novel markers that may be used in the early detection of prediabetes.

## Author contributions

AK: Conceptualization, Formal Analysis, Investigation, Methodology, Writing – original draft, Writing – review & editing. SD-J: Conceptualization, Formal Analysis, Investigation, Methodology, Writing – review & editing.
